# In Situ Underwater Localization of Magnetic Sensors Using Natural Computing Algorithms

**DOI:** 10.3390/s23041797

**Published:** 2023-02-05

**Authors:** Roger Alimi, Elad Fisher, Kanna Nahir

**Affiliations:** 1 Technology Division, Soreq NRC, Yavne 81800, Israel; 2 Hatter Department of Marine Technologies, University of Haifa, Haifa 3498838, Israel

**Keywords:** magnetometers, underwater sensing, genetic algorithm, particle swarm optimization

## Abstract

In the shallow water regime, several positioning methods for locating underwater magnetometers have been investigated. These studies are based on either computer simulations or downscaled laboratory experiments. The magnetic fields created at the sensors’ locations define an inverse problem in which the sensors’ precise coordinates are the unknown variables. This work addresses the issue through (1) a full-scale experimental setup that provides a thorough scientific perspective as well as real-world system validation and (2) a passive ferromagnetic source with (3) an unknown magnetic vector. The latter increases the numeric solution’s complexity. Eight magnetometers are arranged according to a 2.5 × 2.5 m grid. Six meters above, a ferromagnetic object moves according to a well-defined path and velocity. The magnetic field recorded by the network is then analyzed by two natural computing algorithms: the genetic algorithm (GA) and particle swarm optimizer (PSO). Single- and multi-objective versions are run and compared. All the methods performed very well and were able to determine the location of the sensors within a relative error of 1 to 3%. The absolute error lies between 20 and 35 cm for the close and far sensors, respectively. The multi-objective versions performed better.

## 1. Introduction

**Figure 1:**
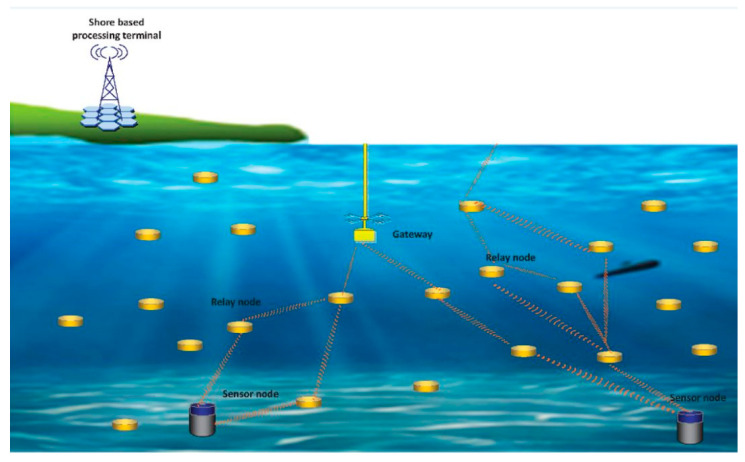
Schematic view of underwater sensor deployment [3].

Underwater sensors arrays have been widely used for various applications such as scientific exploration, sea disaster investigation, and military purposes [1,2,3,4]. Figure 1 shows a schematic view of this kind of deployment.

Acoustic, optical, or RF sensing technologies are commonly used. The challenging task is the precise location estimation for the deployed network, due to the special underwater medium, as well as the lack of GPS data. This is critical to enable the system to provide an accurate description of any investigated phenomenon. Unfortunately, common localization solutions do not provide a satisfactory response to this crucial issue.

In the shallow water regime, solutions for the location of the underwater sensor are often divided into two main categories [5]. Range-based schemes estimate the locations of sensors by using inter-sensor measurements and the prior knowledge of the locations of a few reference sensors [6,7]. Range-free schemes are much simpler, but the location output they supply cannot be very precise [8,9]. Several positioning methods using magnetic technologies have been investigated. The magnetic fields generated at the sensors location, given a known object trajectory, define an inverse problem, in which the precise positions of the sensors are the variables of the equation system.

Callmer et al. [5] utilize triaxial magnetometers and a friendly vessel with a known magnetic dipole to silently localize the sensors. In Yu et al. [10], a solenoid coil carrying a direct current is employed as a magnetic source carried by a boat. This moves along a predetermined trajectory above the sensor field. Using the magnetic measurements, the localization problem is translated into a multi-objective optimization problem. A non-dominated sorting genetic algorithm is used to compute the sensor positions.

Other studies are based on either computer simulations or downscaled laboratory experiments [11,12,13,14,15].

For instance, Bian et al. [11] proposes an oscillating magnetic field-based indoor and underwater positioning system. The magnetic approach generates a bubble-formed magnetic field that is unaffected by environmental variation, unlike radio-wave-based positioning modalities. The proposed system achieves 13.3 cm for the 2D underwater positioning mean accuracy and 19.0 cm for the 3D underwater positioning mean accuracy. In [12], the authors use a magnetic field gradient optimization to localize. The magnetic source in the localization system is a direct current solenoid coil. An objective function is established by measuring the magnetic source’s magnetic field in different positions. A multi-swarm particle swarm optimization with a dynamic learning strategy determines the sensor position vector. Recently, John et al. [13] designed an extensible modular multi-sensor platform prototype that can detect and localize objects with different properties in all environments. Their prototype detects and localizes objects using magnetic, acoustic, and electrical sensors. Although the system is ready for underwater measurements, data fusion algorithms are still being developed.

In [14], the authors derive an approximate equation to calculate the external magnetic field of a three-core armored underwater cable based on seafloor environments and the cable structure. Underwater cable localization uses a dual three-axis magnetic sensor array and beetle swarm optimization (BSO) algorithm. An optimization algorithm replaces analytical geometry, and a magnetic flux-density amplitude fitness function improves underwater cable localization. Although quite promising, the results are only based on simulations. Zhang et al. recently conducted real-world experiments (1.5 m depth in a water tank and 9 m depth in a shallow sea) [15]. An electric field induced by a standard current source is used for real-time underwater equipment location. Stationary or moving underwater equipment is tracked and located in shallow and deep seas (9 m) under noisy conditions. A real-time position is estimated using an extended Kalman filter. Although not based on magnetometers, this system is an interesting competitor despite the fact that it exhibits errors larger than ours (0.5–0.7 m vs. 0.25–0.35 m).

As far as resolution methods are concerned, Philippeaux et al. [16] uses a genetic algorithm. The field tests appear promising after extensive magnetic field modeling and an algorithm simulation. The simulation matches the field tests. Only preliminary in-water testing of the system has been conducted. In [17], Hu et al. present a practical localization algorithm that uses magnetic field vector and gradient tensor data to determine the center coordinates and magnetic moments of multiple underwater magnetic objects. The Levenberg–Marquardt algorithm is used. Hou et al. perform an interesting machine learning approach [18]. They present an Auto Mobile Base Simultaneous localization and mapping (AMB-SLAM) online navigation algorithm based on an artificial neural network (ANN) and measurements from randomly distributed beacons of low-frequency magnetic fields. Their simulations show that a setup of unsophisticated low-cost magnetic beacons can produce geometrically consistent feature maps and an accurate trajectory for AUVs’ underwater navigation.

The present work addresses several issues that have not been addressed previously. First, we construct a full-scale experimental setup that provides comprehensive scientific insight as well as a real-life system validation. In addition, instead of the active coil used in previous studies, we use a passive ferromagnetic source. Finally, the magnetic vector moment of the source is a part of the unknown that the algorithms solved.

The algorithms we have developed provide results that closely match reality in this type of scenario. In order to reproduce more faithfully realistic conditions, we have voluntarily disturbed one of the testing events. Even in this case, the results are still reliable. We also present a comparison between two classes of meta-heuristics, GA and PSO, for which single- and multi-objective implementations are considered.

## 2. Experimental Setup and Methodology

**Figure 2:**
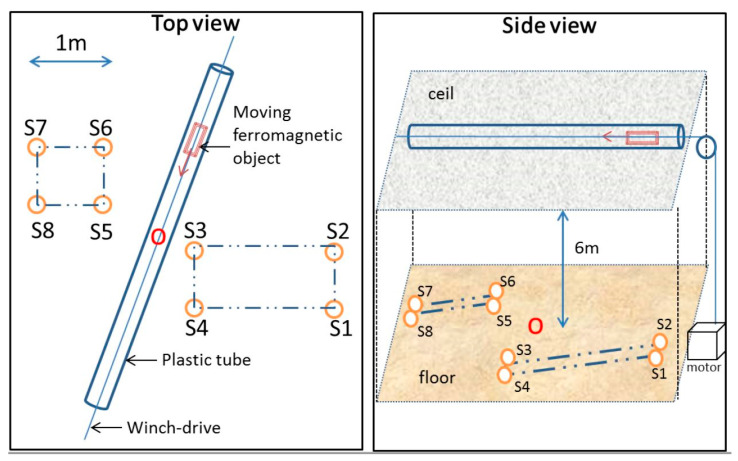
Top and side views of the experimental setup used in the experiment. Note the point O in red, which is the center of the searching box used in both GA and PSO.

Our setup consists of eight vector magnetometers arranged on the ground according to the grid shown in Figure 2. The geometry is chosen to imitate a shallow water deployment in the vicinity of a harbor entrance. Six meters above, a plastic tube is attached on the laboratory ceil. Within the tube, a non-ferromagnetic winch-drive activated by a pulley allows for the motion of the ferromagnetic object. The velocity is motor-controllable from the ground. The only unknown variable remains the moment of the moving object, which we let the algorithms calculate together with the coordinates of the sensors.

Natural computing or nature-inspired algorithms refer to a wide class of computational paradigms in which selected features of natural phenomena or behaviors are studied and imitated to solve complex mathematical systems [19]. Two important subclasses are evolutionary algorithms and swarm intelligence [20]. The former is inspired by the Darwin theory of evolution; the latter is based on emergent properties of the collective behavior of a large number of small, simple agents. Among evolutionary algorithms, the genetic algorithm (GA) is one of the more well-studied ones that has been implemented in a wide variety of applications [21]. Among the swarm intelligence schemes, we choose particle swarm optimization (PSO), which is also a well-known algorithm that has proven itself in a large range of scientific problems [22].

### 2.1. Physical Problem

We assume the dipole approximation of a ferromagnetic object moving on a straight line at constant velocity [23]. This motion deforms the ambient magnetic field recorded by the sensor. The signal shape depends on the trajectory parameters, the magnetic moment of the moving object, and the position of the sensor. The components of the field, $B_{x},B_{y},\;\mathrm{and}\;B_{z}$, are given by Equation (1):

(1) $$\left(\begin{array}{c} B_{x}\\ B_{y}\\ B_{z} \end{array}\right)=\frac{\mu_{0}}{4\pi R^{5}}\left|\begin{array}{ccc} 3x^{2}-R^{2} & 3xy & 3xz\\ 3yx & 3y^{2}-R^{2} & 3yz\\ 3zx & 3zy & 3z^{2}-R^{2} \end{array}\right|\left(\begin{array}{c} M_{x}\\ M_{y}\\ M_{z} \end{array}\right),$$

where $x=x_{sensor}-x_{object}\;$(likewise for *y* and *z*); R is the distance between the sensor and the object; *M_x_, M_y_*, and *M_z_* are the object moment components; and *μ*_0_ is the vacuum magnetic permeability.

In our situation, the geometric parameters of the trajectory are known, including velocity and direction. The sensors’ positions are the main unknowns. Since the moving object is not a controllable magnet or beacon, we cannot assume given values for the moment vector that we must consider as additional variables in the optimization problem. The latter then includes six parameters: three for the sensor position and three for the moment. Since the trajectory is known, there is no other coupling term between the fields measured by the sensors. Hence, each sensor is treated separately.

The algorithm looks for the set of variables that minimizes an error functional expressing the difference between the calculated and measured fields. The proximity of the graphs of two 2D functions is estimated by considering how well the graphs superpose, e.g., evaluate, the cross correlation of the functions. This is achieved using the normalized dot product of the two functions. If P and Q are the curves we compare, the cross-correlation fitness, *f_cc_*, can be written using Equation (2):

(2) $$f_{cc}=\frac{P\;\cdot \;Q}{max\left[P\;\cdot \;P,Q\;\cdot \;Q\right]}\;,$$

where

(3) $$P\;\cdot \;Q=\overset{length\left(P\right)}{\underset{i=1}{\sum }}P_{i}\;\cdot \;Q_{i}.$$

For each sensor, when the errors coming from the three axis curves are summed into one single expression, we have a single-objective scheme. When each sensor axis error is treated separately, we refer to it as multi-objective optimization. The error to minimize is a 3D function of the three axis errors. Although more computationally complex, it has the advantage of benefitting more from the spatial information provided by the vector magnetometer. For both GA and PSO, we have tested both single- and multi-objective versions.

In its most general form, a multi-objective optimization (MO) problem consists of finding, in a set of admissible solutions, a subset of solutions minimizing (or maximizing) its objectives. The main issue in MO is the definition of order (sorting) of two vectors in a space that has the dimension of the number of objectives to optimize. Such a relation is called dominance, and it serves to define a Pareto front. Consider a vector, **f**(**u**), of the decision vector, ***u***, having n variables or objectives. The MO problem can be expressed as minimizing the value of **f** for every variable:

(4) $$\min f\left(u\right)=\left(f_{1}\left(u\right),\;f_{2}\left(u\right),\ldots ,f_{n}\left(u\right)\right)),\;\;u\;\in \;\Omega \;\;$$

Then, **f** can be interpreted as a mapping of the decision space to the objective space. Next, the dominance relation is defined in the decision space (for a minimization problem). Given two vectors, ***u*** and ***v***, ***u*** dominates v if and only if ***f***(***u***) is not larger than ***f***(***v***) for any objective, and it is less for at least one objective. ***u*** and ***v*** are equivalent if and only if ***f***(***u***)and ***f***(***v***) are the same for all objectives; ***u*** and ***v*** cannot be compared if and only if neither dominates the other nor are they not equivalent. The dominance relation is noted ≺ and is formally defined by

$$f\left(u\right)≺f\left(v\right)\;if\;$$

(5) $$\forall i\;\in \left\{\;1,2,\ldots ,n\right\},\;f_{i}\left(u\right)\;\leq \;f_{i}\left(v\right),\;\wedge \exists i,\;f_{i}\left(u\right)<f_{i}\left(v\right)\;.\;$$

Given the dominance relation, the Pareto optimal set, $\rho^{*}$, is defined as the set of all Pareto optimal vectors, where a vector is called optimal if and only if it is not dominated by any other vector of the decision space. Formally speaking:

(6) $$\rho^{*}=\left\{\;u\;\in \;\Omega \;\neg \exists v\;\in \;\Omega ,\;f\left(u\right)≺f\left(v\right)\right\}\;.\;$$

The image set of $\rho^{*}\;$ in the objective space is called a Pareto front:

(7) $$\rho f^{*}=\{f\left(u\right)\;|\;u\;\in \rho^{*}\}\;.\;\;$$

**Figure 3:**
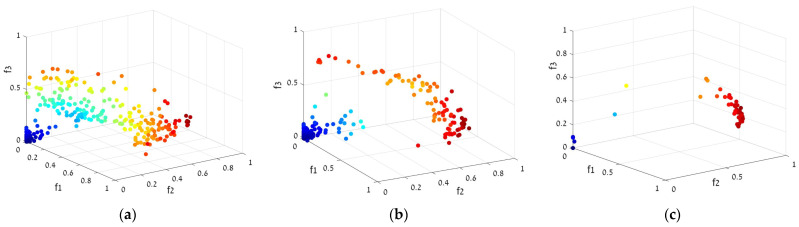
Pareto front dynamics in multi-objective optimization of the genetic algorithm. (**a**–**c**) refer to 5, 15, and 50 generations of the GA, respectively.

Solving the MO problem means finding solutions that are closed to the Pareto front and are uniformly distributed. Figure 3a–c show the Pareto front formation and evolution for one of our Multi Objective Genetic Algorithms (MOGA) runs.

### 2.2. Genetic Algorithm

Genetic algorithms encode potential solutions on a simple chromosome-like structure and apply recombination operators to preserve critical information. They apply the principal concept of “survival of the fittest” on genes achieving best results. An implementation of a genetic algorithm begins with a population of random chromosomes. Each chromosome is a possible solution, which carries six genes, one for each variable of the magnetic equation: three for the sensor position and three for the moment. These structures are evaluated and reproductive opportunities are allocated in such a way that those chromosomes that represent a better solution are given more chances to reproduce than others. Mutation operators are applied to guarantee good exploration of the solution space.

For multi-objective GA, we used a modified version of the fast, elitist Non Selective Genetic Algorithm (NSGAII) algorithm [24]. This algorithm is based on a classification of individuals into several levels. Since, as we shall see later, the NSGAII produces the best solution, we provide a more detailed description of the algorithm here.

The NSGA algorithm does not fundamentally differ from classical genetic algorithms. No choice is imposed on the genetics operators of mutation, crossing, selection, or insertion. The only difference is at the level of the implementation of the assignment operator of the adaptation value of individuals. We have upgraded the classical GA operators by using an adaptive and dynamic mutation mechanism. Moreover, the crossover was modified by adding a simulated annealing moderating process. Finally, we update the size the population according to the convergence of the best fitness values. These three additions greatly increase the convergence rate of the algorithm without deteriorating the quality of the final results.

This assignment operator is based on the rank of the Pareto set to which the solution belongs. In this procedure, all the solutions of the first set are given with the same adaptation value. For all other sets, the adaptation value is equal to the smallest value of the edge solution that precedes it, minus ε, which is a small positive number. This mechanism prevents the situation in which two solutions belonging to two different sets have the same adaptation value. The main disadvantage of this algorithm is the lack of elitism. The NSGA-II algorithm fills this gap.

The presence of elites increases the chances of creating better children, leading to a much faster convergence. In the case of single-criterion optimization problems, the elites are easily identified. They have the best value with respect to the objective function. For multi-criteria optimization problems, this statement is, of course, no longer valid.

In order to manage elites, the NSGA-II algorithm uses the rank of the Pareto set to which the solution belongs. The elites are identified as the solutions that are part of the first Pareto set. To manage diversity at the level of Pareto sets, the algorithm uses a particular implementation of the selection operator called “crowded tournament”. In the crowded distance assignment procedure, the density of the solutions surrounding a given solution are evaluated. This value has the effect of reducing the chances of survival of a solution in a region where several other solutions are concentrated. 

In our scheme, the density is measured in the objective space and not in the decision space. The method directly includes a normalization, which is essential when calculating distances. 

**Figure 4:**
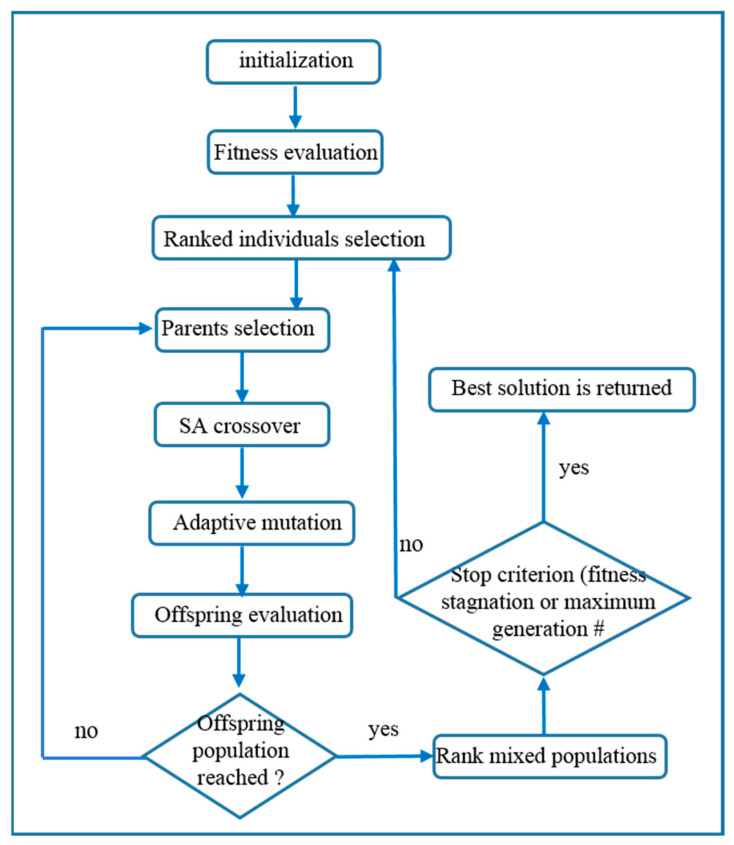
Basic flowchart of the NSGA-II algorithm.

To summarize, the NSGA-II algorithm uses two populations: a parent population P of size N and an offspring population Q, which consists of the set of individuals that have been created by the application of the GA operators. At each iteration, the two populations are combined in an intermediate population, R, and sorted to obtain the Pareto sets. Finally, a new population P’ consists of the best Pareto sets of population R. To achieve this, the solutions of the Pareto sets are included in the population, until the size becomes greater than or equal to the size, N, of the initial population. If, following the addition of the last possible set of Pareto, the population size is greater than N, then the last set is sorted according to the crowded distance, and the solutions with the smallest distances are eliminated until size N is achieved. The selection operator, crossing, and mutation are then applied to P’ to create the new population, Q’, and so on. A basic flowchart of NSGA-II is shown in Figure 4.

GA has already been used as an optimization technique for underwater sensor positioning [25]. However, in that study, GA was employed to calculate the best deployment of the sensor nodes not the exact position of these nodes *after* deployment, as we show in our work. In another important study, already cited above [10], GA was used to compute the position of the sensor, given the known trajectory of a ferromagnetic object like in our study. There are, however, two important differences. First, Yu’s paper reports a laboratory reduced-scale setup, which is more similar to a computer simulation than to a real-world full-scale experiment. Second, the authors use, as the source, a solenoid coil with a given, controllable magnetic moment. This ideal setup reduces, by a factor of 2, the number of unknown parameters (genes in GA terminology) and simplifies the search procedure. We release this constraint by allowing any ferromagnetic object that is big enough to be used, leaving the algorithm to compute, by itself, the source of the magnetic field. Later, we show that this choice does not affect the ability of our GA to successfully determine the exact position of the sensors.

### 2.3. Particle Swarm Optimization

In the PSO paradigm [22], the chromosomes of the GA are replaced by particles moving in the space spanned by the 6 directions defined by the problem’s unknowns (the genes in the GA). From a given position in the space, the new particle direction and amplitude is computed as a weighted combination of its current inertia, its own best past position, and the best past position of the swarm. This is usually understood as a compromise between pure individual and social behaviors.

A particle, *i*, is defined by its position vector, ***x****_i_*, and its velocity vector, ***v****_i_*. In every iteration, each particle changes its position according to the new velocity, see Equations (8) and (9):

(8) $$\;v_{i}^{t+1}=\omega v_{i}^{t}+c_{1}r_{1}\left(xbest_{i}^{t}-x_{i}^{t}\right)+c_{2}r_{2}\left(gbest_{i}^{t}-x_{i}^{t}\right)\;\;,\;$$

(9) $$\;\;\;\;\;\;x_{i}^{t+1}=x_{i}^{t}+v_{i}^{t+1}\;\cdot \;t$$

where *x_best_* and *g_best_* denote the best particle position and best group position, respectively; and the parameters *ω*, *c*_1_, *c*_2_, *r*_1_, and *r*_2_ are inertia weight, two positive constants, and two random parameters within [0, 1], respectively. In the baseline particle swarm optimization algorithm, *ω* is selected as unit, but an improvement of the algorithm is found in its inertial implementation using *ω* ∊ [0.5 0.9]. Usually, maximum and minimum velocity values are also defined, and initially the particles are distributed randomly to encourage the search in all possible locations.

A position is “better” defined according to its fitness value. The fitness function is the same functional error that is defined above. The multi-objective version is inspired by [26]. 

## 3. Results and Discussion

Among the several events (the motion of the target above the grid) performed during the experiments, we selected six of them for testing the algorithms. Events #1, #3, and #6 are the reverse directions of events #2, #4, and #5, respectively. The velocities varied between 0.5 to 1.5 m/s. During event #6, a distractor was moved within the laboratory, creating a degradation of the signal. We see the influence of it in the results. All four algorithms were run: single- and multi-objective GA (NSGA-II) and single- and multi-objective PSO.

Some comments regarding the raw data are required before the experiment results and analysis.

The distance between the sensors and the moving object is at least 6 m. The object dimensions are not longer than 0.5 m in any dimension. In these conditions, we can perfectly assume a dipole approximation for the magnetic field model. In this condition, we know that the magnetic field should decay with a power of 3 of the distance between the sensor and the source. 

The total moment is around 10 Am^2^, as shown later. The magnetic field that the sensor records at a distance of 6 m should be roughly 7 nT [23]. We employ three-axial fluxgate sensors with an internal noise of about 0.25 nT rms (Mag634 by Bartington Instruments Ltd., Oxon OX28 4GG, UK), which is an order of magnitude less than the anticipated signal strength. We calculated an Signal to Noise Ratio (SNR) value of approximately 20, for most events. This is more than sufficient to quantify the magnetic anomaly produced by the moving object.

The sensors’ differences can be seen in two ways: first, in a slight but nevertheless well-discernible difference in the field magnitude; second, in the changes in the phase as a function of time (we use vector magnetometers). These variations enable the localization of each sensor on its own, and our SNR enables a high absolute value precision.

**Figure 5:**
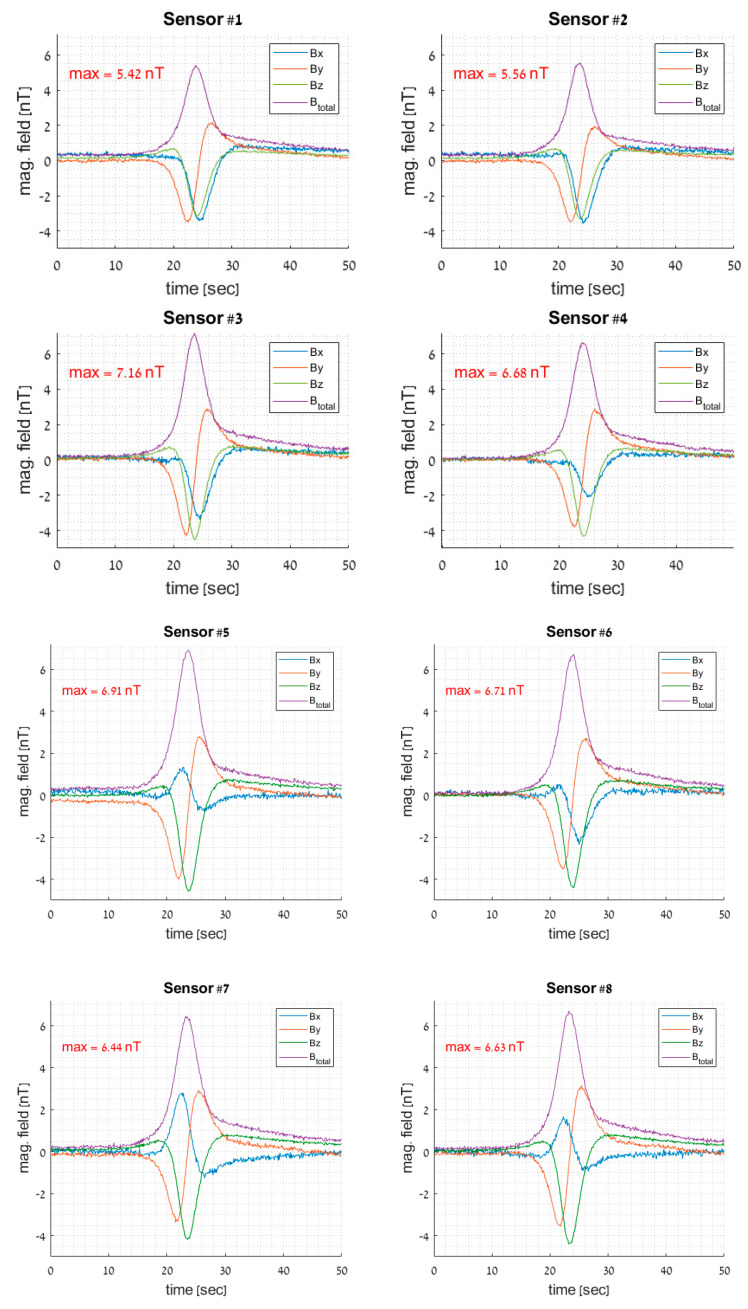
Typical raw data recorded during one of the events.

Figure 5 displays the sensor-measured raw data. Keep in mind that the maximum amplitude values for the near and distant sensors are different: [7.16 6.91 6.71 6.68] nT for sensors #3, #5, #6, and #4, respectively, and [5.42 5.56 6.44 6.63] nT for sensors #1, #2, #7, and #8, respectively. With just an amplitude variation, the sensors on the same side of the trajectory display identical phases throughout each axis. For the sensors located on a separate side of the trajectory, the x phase is the opposite.

Both PSO and GA require a bounded search space. For each sensor, a random point was chosen inside a 5 × 5 m box centered on the point O, as shown in Figure 2. This box has a width of 2 m and is centered on the floor level. The searching space is then defined by a square of 10 m side around this initial point. The algorithm has almost no idea where each sensor is localized when it starts searching.

Both the GA and PSO parameters were optimized using event #1 as the test case (an arbitrary choice). The NSGAII algorithm was slightly modified during the ranking process, and one population from the two generated from the optimal Pareto front was selected. The algorithms were first run 10 times for each of the six events. From these runs, we chose the best result according to the best fitness value. This procedure was repeated 10 times in order to obtain some statistics. We then calculated the weighted error, relative to the confidence of each run, and computed the median error for each coordinate.

**Figure 6:**
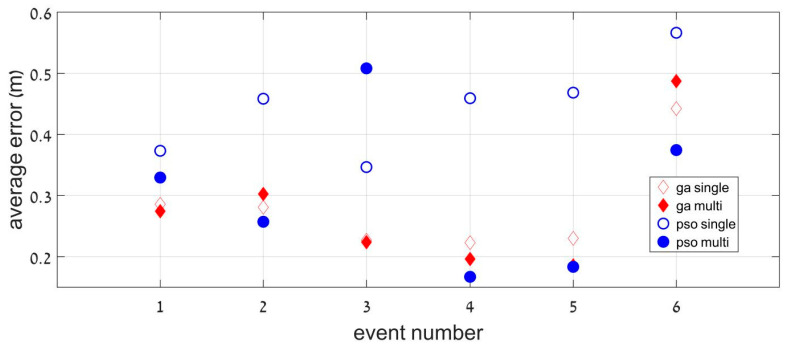
Deviation from exact location (in m) for each event (averaged over the eight sensors).

Figure 6 shows the median error of all the sensors (deviation from their exact position), and Table 1 looks at each sensor averaged over all events. Taken together, Figure 6 and Table 1 compare the four algorithms.

An overall look at Table 1 and Figure 5 shows that the results are excellent. This is particularly encouraging regarding the very large size of the search space. Figure 5 shows that the algorithms were insensitive to the direction, velocity, and moment of the crossing event. Event #6 shows a larger error due to the distractor presence, although its best median location error was only 40 cm. This means that even in more realistic conditions, our method can provide a precise and reliable location of the grid. Overall, the multi-objective optimization is the most appropriate method, either for the GA or PSO algorithm. A remarkably small averaged error (less than 30 cm) was found in both cases.

**Figure 7:**
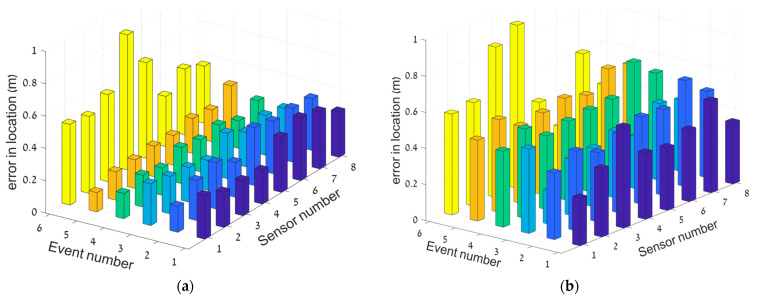
Deviation from exact location (in m) for each event and for each sensor. (**a**) and (**b**) are the best (MOGA) and the worst (SOPSO) results, respectively.

Figure 7 shows a more detailed view of the results, where each sensor’s performance is shown for each event separately. Figure 7a shows the overall best results (obtained with MOGA), while Figure 7b shows the overall worst results (obtained with Single Objective Particle Swarm Optimization (SOPSO)).

The MOGA algorithm performs slightly better that the Multiple Objective Particle Swarm Optimization (MOPSO) algorithm, although the best local solution was obtained using MOPSO; this is encouraging, since it has a smaller number of free parameters. Still, GA looks more stable, probably because of its ability through the mutation operator to exit local minima and find a better optimum. Adding this kind of operator to MOPSO should improve the performance toward errors of less than 20 cm.

As already mentioned, our methods do not suppose a known moment but rather calculate it as a part of the global solution of the inverse problem. The results are shown in Table 2. The values are consistent from sensor to sensor and from event to event, which is a fact that gives us confidence in our calculation schemes. The results from events #1, #3, and #6 show consistently higher values than those from events #2, #4, and #5. This can be expected, since the same moment can have a different projection on the Earth’s magnetic field according to its direction of motion.

## 4. Conclusions

A full-scale experiment was performed that mimics the underwater positioning of magnetic sensors. The position of eight magnetometers was successfully calculated, where the signals generated by a known trajectory of a ferromagnetic object were solved by two kinds of natural computing algorithms. Single- and multi-objective versions of a GA and a PSO were able to compute the position of the sensors, with an error of 25 cm around their exact locations. This is a remarkable result regarding the realistic scale of the setup and the almost blind initial guess locations.

Multi-objective optimization seems to be the most appropriate heuristic technique, despite its higher computation complexity. This is probably because, when looking for the three coordinates of the sensors, the 3D information available in the signals must be taken advantage of in the best way possible. MOGA generally performs better than MOPSO, although the latter sometimes shows better local results. 

Finally, we believe our methods can offer an answer to several open challenges listed in [2], such as reliability, node mobility, and efficiency. Future work includes testing the system in a true maritime environment and using a shallow water deployment of the magnetometers. Several configurations can be considered, from relatively small-scale applications involving securing harbor facilities to larger-scale deployments for monitoring ship motion.

## Figures and Tables

**Table 1:** Deviation from exact location (in m) for each sensor (averaged over the six events).

	GA		PSO	
	Single	Multi	Single	Multi
**Sensor #1**	0.26	0.28	0.44	0.25
**Sensor #2**	0.32	0.29	0.57	0.40
**Sensor #3**	0.29	0.26	0.42	0.32
**Sensor #4**	0.28	0.31	0.44	0.22
**Sensor #5**	0.22	0.21	0.44	0.33
**Sensor #6**	0.25	0.22	0.42	0.17
**Sensor #7**	0.23	0.22	0.47	0.25
**Sensor #8**	0.24	0.21	0.43	0.24

**Table 2:** Calculated total moments (A m^2^) using the MOGA method.

		Sensor #							
		1	2	3	4	5	6	7	8
**Event #**	**1**	9.05	9.35	8.42	8.69	9.55	9.61	9.05	**9.04**
	**2**	8.64	7.9	8.22	8.18	8.02	8.15	8.25	**7.73**
	**3**	9.23	9.02	9.18	8.59	8.17	8.52	8.68	**8.89**
	**4**	7.89	8.17	8.25	8.21	7.75	7.87	7.96	**7.88**
	**5**	8.4	8.08	7.86	8.01	7.96	8.18	8.12	**7.82**
	**6**	9.21	8.84	9.25	8.76	8.48	9.01	9.16	**9.4**

## Data Availability

The data presented in this study are available on request from the corresponding author. The data are not publicly available due to legal restrictions.
